# Development and Characterization of Novel Site Specific Hollow Floating Microspheres Bearing 5-Fu for Stomach Targeting

**DOI:** 10.1155/2014/705259

**Published:** 2014-10-14

**Authors:** Peeyush Bhardwaj, Deepti Chaurasia, Ranjit Singh, Anoop Swarup

**Affiliations:** ^1^Institute of Pharmacy, Bundelkhand University, Jhansi, Uttar Pradesh, India; ^2^NKBR College of Pharmacy & Research Centre, Meerut, Uttar Pradesh, India; ^3^Government College of Pharmacy, Rohru, Shimla, Himachal Pradesh, India; ^4^Jagran Lakecity University, Bhopal, Madhya Pradesh, India

## Abstract

Multiple-unit-type oral floating hollow microspheres of 5-fluorouracil (5-Fu) were developed using modified solvent evaporation technique to prolong gastric residence time, to target stomach cancer, and to increase drug bioavailability. The prepared microspheres were characterized for micromeritic properties, floating behavior, entrapment efficiency, and scanning electron microscopy (SEM). The *in vitro* drug release and floating behavior were studied in simulated gastric fluid (SGF) at pH 1.2. The yield of microspheres was obtained up to 84.46 ± 6.47%. Microspheres showed passable flow properties. Based on optical microscopy, particle size was found to be ranging from 158.65 ± 12.02 to 198.67 ± 17.45 *μ*m. SEM confirmed spherical size, perforated smooth surface, and a hollow cavity inside the microspheres. Different kinetic models for drug release were also applied on selected batches.

## 1. Introduction

Oral route of administration is the most convenient and widely used method of drug administration. And the development of the stomach specific oral controlled-release drug delivery system is a challenging job because of the variation of pH in different segments of the gastrointestinal (GI) tract, the fluctuation in gastric emptying time, and the difficulty of localizing an oral delivery system in a selected region of the GI tract. Rapid GI transit can prevent the absorption of complete drug in the absorption zone and reduce the effectiveness of the administered dose since the majority of drugs are absorbed in stomach or the upper part of the small intestine [1, 2].

To overcome the above discussed issues, many types of oral controlled drug delivery systems having prolonged gastric residence times have been reported like floating drug delivery systems (FDDS) [3–7], swelling or expanding system [8], mucoadhesive systems [9, 10], modified-shape systems [11], high-density systems [12], and other delayed gastric emptying devices.

FDDS has lower density than gastric fluids and thus remains buoyant in the stomach fluid without affecting the gastric emptying for a prolonged period of time. While the system is floating in the gastric fluid, the drug is released slowly from the system at a desired rate. Materials used for FDDS include carbon dioxide forming agents (carbonate or bicarbonate compounds) [8, 13], highly swellable hydrocolloids, and light mineral oils [14, 15]. Multiple unit systems [12, 16, 17] and hollow systems prepared by solvent evaporation methods [18–20] have also been reported.

The best medium for assessment of* in vitro* floating ability is 0.1 N HCl containing 0.02% tween 20. Tween 20 is added to counteract the downward pulling at the liquid surface by lowering surface tension of the medium [18].

However, it has been shown that products based on multiple unit systems comprising many small units have advantages over single-unit preparations such as matrix tablets [21]. The gastric emptying of multiple unit dosage forms occurs gradually, in a more consistent manner, with less individual variation [22, 23]. Multiple unit dosage forms also have the potential to distribute extensively over a large area in the stomach and small intestine, thus producing a more predictable drug release by suppressing the effect of many variables in the gastrointestinal environment. As multiple unit dosage forms consist of many small units, less risk of dosage dumping is expected [24].

5-Fluorouracil (5-Fu) is an antimetabolite of the pyrimidine analog class, which is widely used alone or in combination with chemotherapy regimens in a variety of solid cancers, such as stomach, colon, lung, and breast cancer.

It interferes with nucleic acid synthesis, inhibits DNA synthesis, and eventually inhibits cell growth [25]. However, 5-Fu may cause bone marrow depression, gastrointestinal tract reaction, or even leukopenia and thrombocytopenia. It has also been recognized that, optimally, this drug should be dosed once or twice a week, preferably a long-acting injectable and targeted to the desired sites. Because of the short plasma half-life of 10–20 min, high doses, for example, 400–600 mg/m^2^, have to be administered weekly to reach a therapeutic drug level. It is poorly absorbed after oral administration with extremely variable bioavailability [26].

The above-discussed disadvantages make this drug a relevant candidate for microencapsulation. Floating hollow microspheres are one of the multiparticulate delivery systems and are prepared to obtain prolonged or controlled drug delivery to improve bioavailability and to target drug to specific sites. Microspheres can also offer benefits like limiting fluctuation within the therapeutic range, reducing site effects, decreasing dosing frequency, and improving patient compliance [27].

In the present study, the multiple-unit-type oral floating hollow microspheres bearing 5-Fu were developed by modified solvent evaporation technique using Eudragit S-100 as polymer. This polymer is soluble in intestinal fluid; hence, it was used as a carrier to maintain its integrity in the acidic medium of the stomach for a prolonged period of time. Generally, the Higuchi model describes the release of the drug from such insoluble matrix [28].

The high surface tension of the stirring phase causes the solidification and aggregation of polymer on the surface when the polymeric solution is poured from upside. To minimize the contact of polymer solution with the interface, a new method of introducing the polymer solution into the stirring phase was adopted as established by Lee et al. [29].

Formulation was optimized by changing the ratio of the polymer to get the spherical shape with the desired features. The effects of polymer concentration on the yield, particle size distribution, encapsulation efficiency, surface properties, and 5-Fu release rate from hollow microspheres were examined. The prepared spherical hollow microspheres were also evaluated for micromeritic properties, drug content, and SEM as well as for* in vitro* drug release studies.

## 2. Materials and Methods

5-fluorouracil was obtained as a gift sample from Intas Pharmaceutical Ltd., Gujarat. Eudragit S-100 (Rohm Pharma GmbH, Germany) was used as a polymer. Light liquid paraffin (Central Drug House Pvt. Ltd., New Delhi) served as a nonaqueous dispersion phase. All other chemicals and reagents were of analytical grade and were used without further purification.

### 2.1. Preparation of Hollow Microspheres

**Figure 1:**
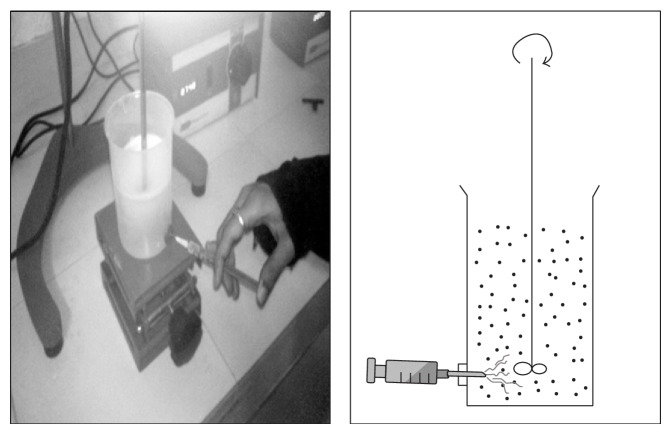
Method of injection of volatile phase into liquid paraffin.

Floating hollow microspheres were prepared by the method reported by Kawashima et al. with slight modifications [19]. Nine formulations of hollow microspheres were developed. Different ratios of 5-Fu and Eudragit S-100 were mixed in a mixture of acetone (ACTN) and ethanol (ETN) (Table 1). The resulting suspension was added slowly with stirring to 250 mL light liquid paraffin at room temperature from the bottom side as shown in Figure 1 [29]. The stirring was continued for two hours at the specified rpm by the mechanical stirrer equipped with four blade propellers in order to evaporate the solvent. After evaporation of solvent, microspheres were collected by filtration, washed repeatedly with petroleum ether until free from oil. The collected microspheres were dried at room temperature and stored in a desiccator.

### 2.2. Characterization of Floating Microspheres

#### 2.2.1. Morphology

**Figure 2:**
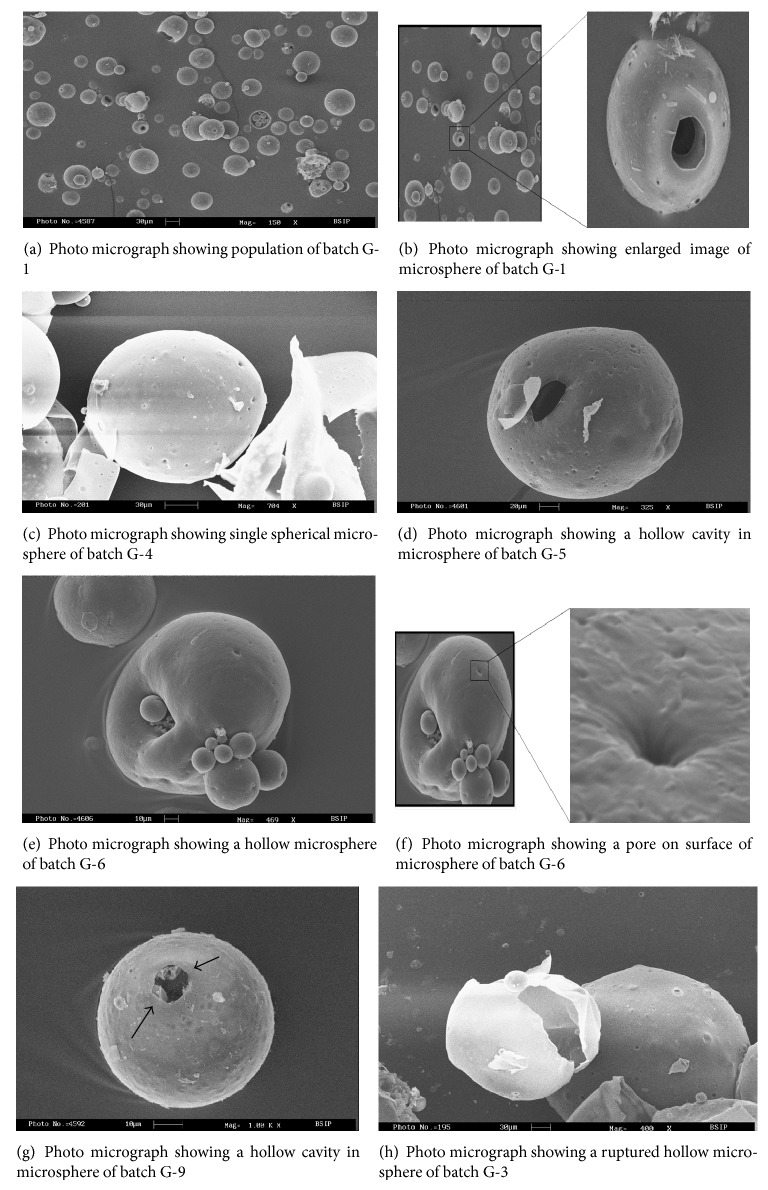
Scanning electron micrograph of floating hollow microsphere.

The morphology of microspheres was studied by scanning electron microscopy (SEM). The samples for SEM were prepared by sprinkling the microspheres on a side of adhesive tape stuck to a stub. Gold palladium coating the prepared stub was done using sputter coater (POLARON model SC-76430). The thickness of coating was 200Å. The coated stubs were randomly scanned under electron microscope (LEO-430, UK). The photomicrographs of the prepared microspheres are presented in Figures 2(a) to 2(h).

#### 2.2.2. Particle Size Analysis

The size of microspheres was determined using an optical microscope (Magnus MLX-DX, Olympus, India) fitted with an ocular micrometer and a stage micrometer. The mean particle size was calculated by measuring 200–300 particles (Table 2).

#### 2.2.3. Micromeritic Properties

The prepared microspheres were characterized for their micromeritic properties such as true density, tapped density, and %compressibility index. The tapping method was adopted to calculate the tapped densities and %compressibility index using following equations [30] (Table 2):

(1) $$\begin{array}{c} \text{Tapped}\;\text{Density}=\frac{\text{Mass}\;\text{of}\;\text{Microspheres}}{\text{Volume}\;\text{of}\;\text{Microspheres}\;\text{after}\;\text{tapping}}, \end{array}$$

(2) $$\begin{array}{c} \%\;\text{Compressibility}\;\text{Index}=\left(\frac{1-V}{V_{O}}\right)\times 100. \end{array}$$

Here, *V* and *V*_*O*_ are the volumes of the samples after and before the standard tapping, respectively.

True density of hollow microspheres was determined by immersing the microparticles in 0.02% tween 80 solution for three days in a metal mesh basket. The microparticles that were sunk after the process were used for density measurement as carried out by the displacement method [31].

#### 2.2.4. Angle of Repose

The flow characteristics, such as the angle of repose (*θ*) of the microspheres, which measures the resistance to particle flow, were determined by fixed funnel method using the following equation [30] (Table 2):

(3) $$\begin{array}{c} \text{Tan}\theta =\frac{h}{r}, \end{array}$$

where, *h* = height of pile and *r* = radius of the base of the pile on the graph paper.

#### 2.2.5. Yield of Microspheres

The prepared microspheres were collected and weighed. The actual weight of the obtained microspheres was divided by the total amount of all nonvolatile material that was used for the preparation of the microspheres [32] (Table 3):

(4) $$\begin{array}{c} \%\;\text{Yield}=\frac{\text{Actual}\;\text{weight}\;\text{of}\;\text{the}\;\text{product}}{\text{Total}\;\text{weight}\;\text{of}\;\text{the}\;\text{excipients}\;\text{and}\;\text{drug}}\times 100. \end{array}$$

#### 2.2.6. Incorporation Efficiency

To determine the incorporation efficiency, 50 mg microspheres were taken and dissolved in 25 mL of methanol. The solution was filtered to separate shell fragments. The estimation of drug was carried out at the *λ*_max⁡_ of 266 nm using a UV double-beam spectrophotometer (Shimadzu UV-1700 series). The incorporation efficiency was calculated using the following equation [32] (Table 3):

(5) Incorporation  Efficiency=Calculated  drug  contentTheoretical  drug  content ×100.

#### 2.2.7. *In Vitro* Floating Ability

**Figure 3:**
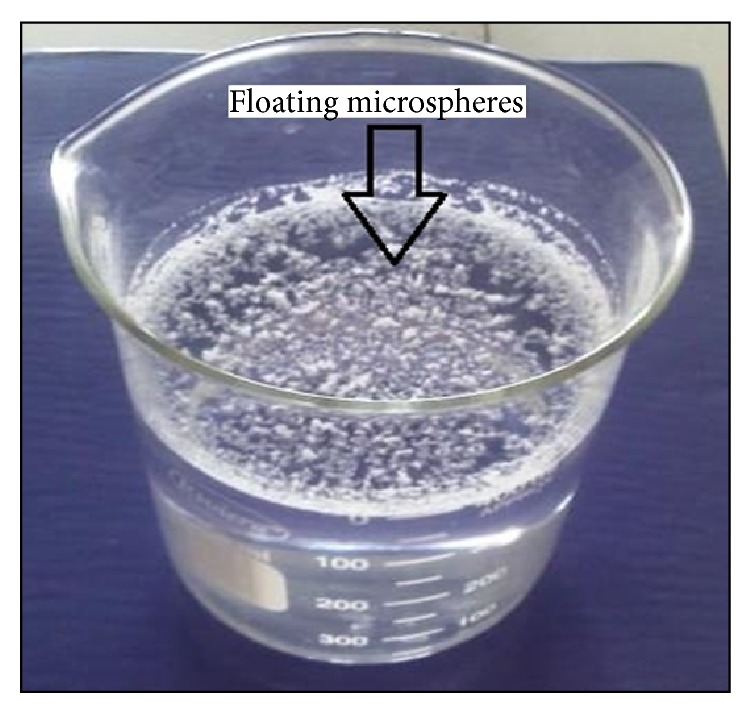
Floating hollow microspheres.

50 mg of floating microspheres were placed in 50 mL beaker. 20 mL of 0.1 N HCl containing 0.02% tween 20 was added to that. The beaker was shaken horizontally in a water bath at 37 ± 0.1°C. Floated particles were collected after 10 hours and dried in a desiccator until constant weight. This process was applied to all the batches. Floating hollow microspheres are shown in Figure 3. The percentage of floating microspheres was calculated using the following equation [32] (Table 3):

(6) % Floating  Ability=Weight  of  floating  microspheresInitial  weight  of  Microspheres ×100.

### 2.3. *In Vitro* Drug Release

*In vitro* drug release studies were performed in 0.1 N HCl (pH 1.2) for floating hollow microspheres. The drug release rate of the floating hollow microspheres was determined in 900 mL 0.1 N HCl at 100 rpm by using USP XXIII dissolution apparatus (paddle type). A weighed amount of floating hollow microspheres equivalent to 50 mg drug was placed in a nonreacting muslin cloth having a smaller mesh size than the microspheres. The mesh was tied with a nylon thread to avoid the escape of any microspheres, and a glass bead was placed in the mesh to induce sinking of microspheres in the dissolution medium [18]. The temperature of the dissolution medium was maintained at 37 ± 0.5°C.

**Figure 4:**
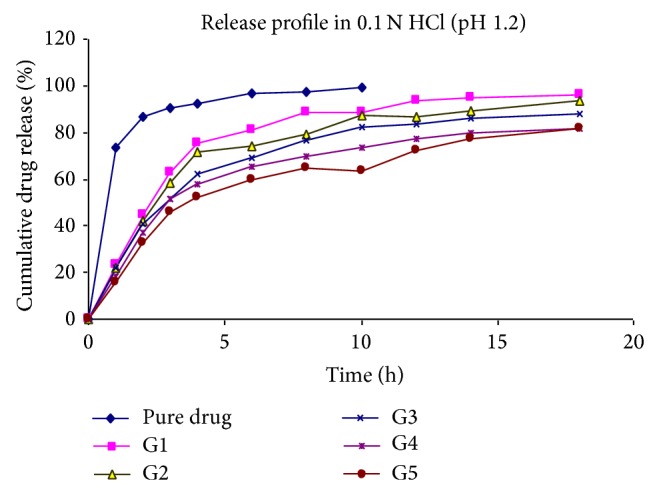
Effect of polymer ratio on release profile of drug in 0.1 N HCl.

**Figure 5:**
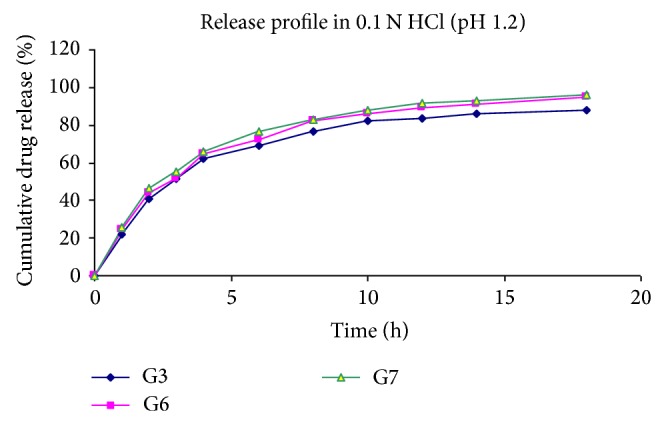
Effect of stirring rate on release profile of drug in 0.1 N HCl.

**Figure 6:**
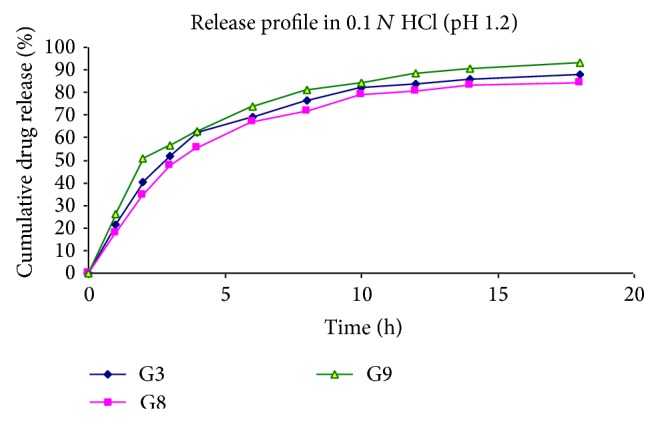
Effect of solvent ratio on release profile of 5-Fu in 0.1 N HCl.

At specified time intervals, 5 mL aliquots were withdrawn, filtered, diluted with the same medium, and assayed at 266 nm for 5-Fu using a UV double-beam spectrophotometer (Shimadzu UV-1700 series). Samples withdrawn were replaced with equal volume of the same dissolution medium. All the experiments as specified above were conducted in triplicate (Tables 4, 5, and 6, Figures 4, 5, and 6).

### 2.4. Kinetics of Drug Release

The zero-order rate (see, (7)) describes systems where the drug release is independent of its concentration, and this is applicable to the dosage forms like transdermal system, coated forms, and osmotic system as well as matrix tablets with low soluble drugs. The first-order equation (8) describes systems in which the release is dependent on its concentration (generally seen for water-soluble drugs in the porous matrix). The Higuchi model describes the release of the drug from an insoluble matrix to be linearly related to the square root of time and is based on Fickian diffusion (see, (9)). The Hixson-Crowell cube root law (see (10)) describes the release of drug from systems where it depends on the change in the surface area and the diameter of the particles or tablets with time and mainly applies in the case of systems that dissolute or erode over time. In order to authenticate the release model, dissolution data can further be analyzed by Peppas and Korsmeyer equation (see, (11)):

(7) $$\begin{array}{c} Q_{t}=k_{n}t, \end{array}$$

(8) $$\begin{array}{c} \ln Q_{t}=\ln Q_{0}-k_{1}t, \end{array}$$

(9) $$\begin{array}{c} Q_{t}=k_{\text{HC}}t^{1/2}, \end{array}$$

(10) $$\begin{array}{c} Q_{0}^{1/3}-Q^{1/3}=k_{\text{HC}}t, \end{array}$$

(11) $$\begin{array}{c} \frac{M_{t}}{M_{\infty }}=kt^{n}, \end{array}$$

where *Q*_*t*_ is the amount of drug released at time *t*; *Q*_0_ is the initial amount of the drug in the formulation; *k*_0_, *k*_1_, *k*_H_, and *k*_HC_ are release rate constants for zero-order, first-order, Higuchi model, and Hixson-Crowell rate equations. In (11), *M*_*t*_ is the amount of drug released at time *t*, and *M*_*∞*_ is the amount released at time *∞*; *k* is the kinetic constant, and *n* is the diffusion coefficient [28].

The release data of various formulations were fitted in various models to ascertain the mechanism of drug release. The observations for the drug release are recorded in Table 7.

### 2.5. Preparation of Dosage Form for* In Vivo* Studies

The optimized formulations, which showed good* in vitro* buoyancy and sustained-release behavior, were finally selected for an* in vivo* study (i.e., radiography). The drug in all selected formulations was replaced with the same amount of barium sulphate while all other ingredients were kept constant. These formulations were analyzed for their physical properties. The analysis confirmed that the developed dosage forms were similar to those containing drug.

#### 2.5.1. *In Vivo* Studies

The experimental protocol to carry out* in vivo* radiographic studies was reviewed and approved by the Institutional Animal Ethical Committee of Shobhit University, India (registration number 1279/ac/09/CPCSEA). The* in vivo* radiographic studies were conducted in young and healthy male albino rabbits weighing 2.0 to 2.2 kg. The animals were kept under standard laboratory conditions (temperature 25 ± 2°C). Rabbits were kept for one week in the animal house to acclimatize them and were fed a fixed standard diet. The 4 healthy male albino rabbits were used to monitor the* in vivo* transit behavior of the prepared floating hollow microspheres. None of the animals had symptoms or history of gastrointestinal (GI) disease. In order to standardize the conditions of GI motility, the animals were fasted for 12 hours prior to the commencement of each experiment. In each experiment, the first radiographic image of the animal subjects was taken to ensure the absence of radioopaque material in the GIT. One of each dosage form prepared for radiography was orally administered to rabbits with the sufficient amount of water. During the study, the rabbits were not allowed to eat, but water was available* ad libitum* [33, 34].

**Figure 7:**
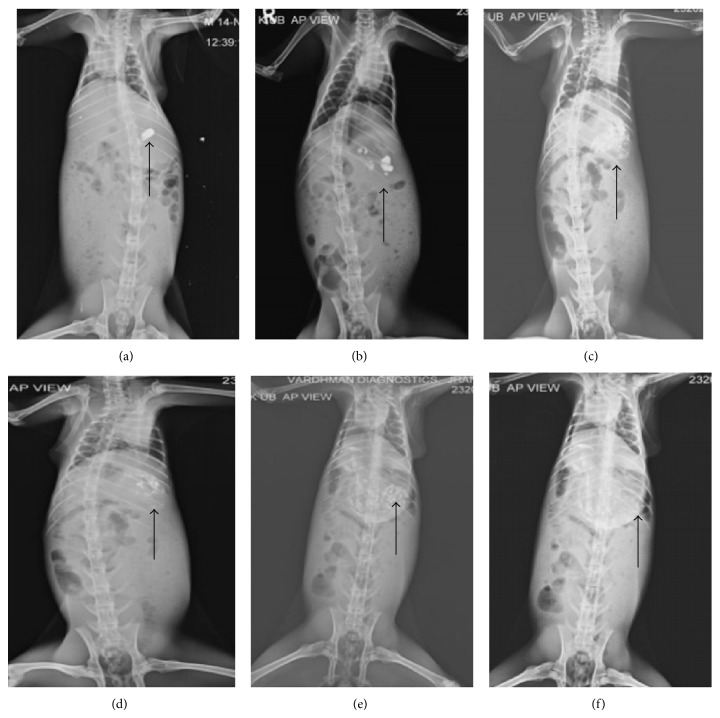
Radiographic images showing the presence of BaSO_4_ loaded floating hollow microspheres in the stomach at different time intervals. Images were taken after (a) 0 hr, (b) 1 hr, (c) 2 hr, (d) 3 hr, (e) 4 hr, and (f) 6 hr.

For radiographic imaging, the legs of the rabbit were tied over a piece of plywood (20 × 20 inch), and location of the formulation in the stomach was monitored by keeping the subjects in front of X-ray machine (Allengers, Bharat Electrical, India, model number E-080743). The distance between the source of X-rays and the object was kept the same during the imaging process. Gastric radiography was done at the intervals of 30 min, 1 hr, 2 hr, 3 hr, 4 hr, 5 hr, and 6 hr. In between the radiographic imaging, the animals were freed and allowed to move and carry out normal activities but were not allowed to take any food (Figure 7).

## 3. Results and Discussions

The formulations of 5-Fu loaded hollow floating microspheres were developed by modified nonaqueous solvent evaporation method. In this method, the drug-polymeric suspension was introduced into stirring liquid paraffin from the bottom side to reduce the aggregation of polymer on the top of liquid paraffin as shown in Figure 1. This technique resulted in spherical microspheres and good yield of product could be achieved.

**Figure 8:**
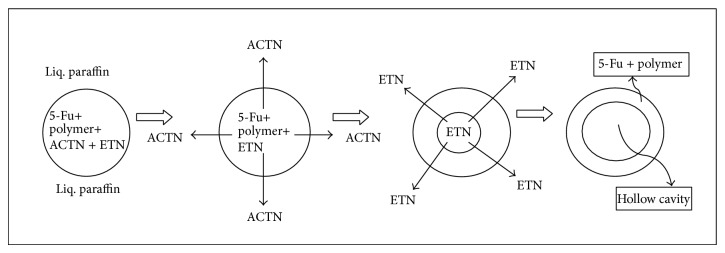
Mechanism of formation of floating hollow microspheres.

The formulation mechanism (Figure 8) of polymeric hollow microspheres is well documented in the literature. The polymer and drug were dissolved in the solvent mixture, such as acetone and ethanol. The polymer solution/suspension is emulsified in continuous phase. Then, the acetone diffused out of the embryonic microspheres into continuous phase. On the other hand, the ethanol, which could not diffuse, is retained in the microspheres as core material. On keeping these microspheres at 40°C, the vapors of alcohol are generated and escaped, leaving a hollow cavity within the microspheres. This hollow cavity provides the ability of buoyancy to the microspheres.

SEM photo micrograph confirmed that the prepared microspheres were spherical with smooth perforated surface (Figures 2(a)–2(h). The formation of perforation may be attributed to the evaporation of alcohol form embryonic microspheres. These images also confirmed that rapid evaporation of ethanol may cause rapture of microspheres (Figure 2(h)). The prepared microspheres were found to be spherical in shape with smooth perforated surface as indicated by photomicrographs. Hollow cavities could also be observed on some micrographs (Figure 2(g)).

The mean particle size of the floating hollow microspheres was found to be ranging from 158.65 ± 12.02 *μ*m to 198.67 ± 17.45 *μ*m. It was observed that, on increasing the polymer amount, the average particle size increased. This may be due to diminished shearing efficiency at higher concentration of the polymer (higher viscosity). It was also observed that, on increasing the volume of ethanol, average particle size was found to increase.

The measured tapped density was found to be in the range of 0.177 ± 0.012 to 0.293 ± 0.011 g/cm^3^. Compressibility index ranged from 7.33 ± 1.15 to 20.00 ± 2.00. The true density of the microspheres was found to be in the range of 0.78 ± 0.04 to 1.08 ± 0.18 g/cm^3^.

The angle of repose was found to be in the range of 31.20 ± 2.05 to 44.12 ± 1.14°. All the batches showed good flow properties except bathes G-1 and G-6, which exhibited higher angle of repose. The %yield of microspheres was found between 84.46 ± 6.47 and 63.21 ± 1.91, which seemed to be good because, in the adopted method of preparation of microspheres, the polymeric-drug suspension was injected into stirring liquid paraffin from bottom side, which resulted in less aggregate formation of polymers on the surface of the liquid paraffin.

The drug entrapment/incorporation efficiency of microspheres was found to be satisfactory (from 54.78 ± 1.15 to 73.67 ± 3.15%). All hollow microspheres showed good floating ability/buoyancy (from 64.60 ± 2.42 to 82.80 ± 3.10%) up to 18 hours with zero lag time. This could be attributed to the insolubility of the used polymers in the gastric fluid. It was observed that the floating ability increased with increasing average particle size.

It was also observed that the formulation prepared with higher volume of ethanol (batch G-9) showed better floating ability compared to other batches. The reason behind this may be the formation of larger air core, which made them less dense than those of gastric fluid.

The release of 5-Fu from the developed floating hollow microspheres was studied in 0.1 N HCl (pH 1.2) up to 18 hours. Since the acrylic polymers are insoluble in the acidic medium, the microspheres retained their integrity during the* in vitro* dissolution studies. The cumulative drug release for microspheres was found to be between 81 and 96%.

It was noted that, on increasing the polymer ratio, the cumulative %drug release decreased gradually. It may be because of increased partitioning of drug in polymer (Eudragit S-100). It was also observed that the release of 5-Fu decreased significantly (*P* < 0.05) on increasing the amount of polymers.

Further, a significant increase was found in drug release on increasing the stirring rate. It may be because of increasing surface area of microspheres due to reduced size.

On increasing the volume of acetone in solvent phase, the release was found to be increased. On the other hand, when the volume of ethanol was increased, the release of drug was found to be decreased. The reason behind this may be the size of microspheres, which increases *n*, increasing the volume of ethanol.

The release profile data of all the developed formulations were fitted in kinetic model using PCP Disso software. The best-fit model was selected on the basis of the values of the residual sum of squares (RSS).

The drug release of batches G-3 and G-9 showed Peppas Korsmayer model; that of batches G-2, G-4, G-5, and G-8 showed the matrix model of drug release; while that of batches G-1, G-6, and G-7 showed first-order of drug release. All batches except for G-5 released the drug in Fikian manner.

The hard gelatin capsule containing BaSO_4_ loaded hollow floating microspheres was clearly visible in the stomach after oral administration of dosage form. In the radiographic image taken after one hour, all microspheres were found to be scattered in the stomach. Dense images of microspheres were seen at initial hours, but, as time passed on, the images of microspheres became lighter. It may be because of the distribution and scattering of microspheres within GI region. The radiographic images indicted that these hollow floating microspheres were retained successfully in the stomach up to six hours.

## 4. Conclusion

Novel floating hollow microspheres were successfully prepared by solvent evaporation method with the modified techniques of administration of polymeric solution/slurry from bottom side, for prolonged as well as stomach specific action of 5-FU. Due to its low density, this multiparticulate drug delivery system showed good floating ability. From* in vitro* drug release studies, it can be concluded that, by changing the ratio of polymer and solvent, drug release can be controlled.* In vivo*, X-ray radiographic studies also confirmed that the prepared dosage forms are able to be retained in GIT for a prolonged period of time.

## Figures and Tables

**Table 1:** Batch specifications for floating hollow microsphres.

Sl. Number	Batch	Drug polymer ratio (5 Fu : Eudragit S-100)	Stirring rate (RPM)	Solvent ratio (ETN : ACTN)
1	G-1	1 : 1	900	1 : 1
2	G-2	1 : 2	900	1 : 1
3	G-3	1 : 3	900	1 : 1
4	G-4	1 : 4	900	1 : 1
5	G-5	1 : 5	900	1 : 1
6	G-6	1 : 3	1100	1 : 1
7	G-7	1 : 3	1300	1 : 1
8	G-8	1 : 3	1200	1 : 3
9	G-9	1 : 3	1400	3 : 1

**Table 2:** Micromeritic properties of floating hollow microspheres.

Batch code	Mean particle size^a^ (*μ*m)	True density^b^ (g/cm^3^)	Tapped density^b^ (gm/cm^3^)	Compressibility index^b^ (%)	Angle of repose^b^ (*θ*)
G-1	158.65 ± 12.02	0.79 ± 0.01	0.177 ± 0.012	18.67 ± 3.06	44.12 ± 1.14
G-2	163.46 ± 13.48	0.78 ± 0.04	0.180 ± 0.017	16.00 ± 2.00	36.42 ± 2.42
G-3	187.99 ± 13.38	0.90 ± 0.04	0.217 ± 0.011	20.00 ± 2.00	35.41 ± 1.44
G-4	198.67 ± 17.45	0.89 ± 0.03	0.2533 ± 0.012	13.33 ± 1.15	33.93 ± 1.47
G-5	195.24 ± 17.88	0.87 ± 0.03	0.223 ± 0.015	11.33 ± 2.31	35.07 ± 4.89
G-6	176.56 ± 11.22	0.83 ± 0.02	0.200 ± .020	14.67 ± 3.06	40.37 ± 3.37
G-7	167.07 ± 19.61	0.93 ± 0.01	0.280 ± 0.010	16.00 ± 2.00	33.02 ± 1.24
G-8	168.68 ± 23.05	0.94 ± 0.03	0.270 ± 0.010	8.67 ± 3.06	34.17 ± 0.42
G-9	196.34 ± 25.21	1.08 ± 0.18	0.293 ± 0.011	7.33 ± 1.15	31.20 ± 2.05

^a^Mean SD, *n* = 200–300.

^b^Mean SD, *n* = 3.

**Table 3:** Various formulation parameters of floating hollow microspheres.

Batch code	Yield^a^ (%)	Incorporation efficiency^a^ (%)	Buoyancy^a^ (%)
G-1	70.12 ± 3.15	54.78 ± 1.15	64.6 ± 2.42
G-2	76.95 ± 0.79	58.00 ± 2.00	71.67 ± 3.52
G-3	84.46 ± 6.47	63.42 ± 3.15	72.40 ± 3.60
G-4	63.21 ± 1.91	67.03 ± 7.09	78.66 ± 1.89
G-5	83.38 ± 2.99	74.30 ± 4.04	80.53 ± 3.50
G-6	73.22 ± 10.57	59.50 ± 2.50	66.53 ± 3.61
G-7	80.34 ± 1.83	63.04 ± 1.73	71.06 ± 4.10
G-8	83.08 ± 3.01	68.79 ± 3.89	71.93 ± 2.10
G-9	66.22 ± 4.93	73.67 ± 3.15	82.80 ± 3.10

^a^Mean SD, *n* = 3.

**Table 4:** Effect of polymer ratio on drug release in 0.1 N HCl.

Sl. No.	Time (hrs)	Cumulative % drug release					
		Pure drug	G1	G2	G3	G4	G5
1	0	0.00 ± 0.00	0.00 ± 0.00	0.00 ± 0.00	0.00 ± 0.00	0.00 ± 0.00	0.00 ± 0.00
2	1	73.47 ± 0.12	23.27 ± 1.77	21.71 ± 2.11	21.71 ± 2.89	17.97 ± 1.89	15.48 ± 1.48
3	2	86.97 ± 0.75	44.60 ± 2.15	42.10 ± 2.67	40.54 ± 2.12	37.09 ± 2.75	32.406 ± 2.67
4	3	90.26 ± 0.46	62.93 ± 3.99	58.552 ± 1.97	51.68 ± 3.67	51.64 ± 2.89	45.99 ± 0.32
5	4	92.63 ± 0.50	75.135 ± 4.66	71.65 ± 3.21	62.25 ± 1.90	57.54 ± 1.82	51.85 ± 1.78
6	6	96.56 ± 0.25	80.84 ± 2.56	74.23 ± 2.78	69.14 ± 0.99	65.02 ± 2.66	59.62 ± 3.11
7	8	97.40 ± 0.99	88.77 ± 4.34	79.31 ± 2.88	76.38 ± 3.10	69.75 ± 2.10	64.63 ± 1.56
8	10	99.18 ± 0.13	88.63 ± 2.51	87.23 ± 2.11	82.10 ± 2.98	73.24 ± 2.57	63.73 ± 1.77
9	12	—	93.48 ± 2.56	86.46 ± 3.11	83.79 ± 2.71	77.07 ± 3.78	72.50 ± 3.67
10	14	—	94.92 ± 2.58	89.11 ± 2.18	86.12 ± 2.88	79.98 ± 3.11	77.25 ± 2.89
11	18	—	96.36 ± 1.89	93.33 ± 2.89	88.14 ± 3.78	81.66 ± 4.22	81.41 ± 2.12

Mean ± SD, *n* = 3.

**Table 5:** Effect of stirring rate on drug release in 0.1 N HCl.

Sl. number	Time (hrs)	Cumulative % drug release		
		G3	G6	G7
1	0	0.00 ± 0.00	0.00 ± 0.00	0.00 ± 0.00
2	1	21.71 ± 1.68	24.21 ± 2.45	25.46 ± 2.32
3	2	40.54 ± 1.65	43.99 ± 2.56	46.18 ± 3.22
4	3	51.68 ± 2.47	51.71 ± 1.66	55.16 ± 1.67
5	4	62.25 ± 3.79	64.47 ± 1.45	66.07 ± 3.27
6	6	69.14 ± 1.94	71.99 ± 2.66	76.723 ± 1.85
7	8	76.38 ± 2.45	82.05 ± 1.78	82.75 ± 2.66
8	10	82.10 ± 1.79	86.24 ± 2.92	88.19 ± 1.49
9	12	83.79 ± 1.93	89.52 ± 2.89	91.48 ± 2.67
10	14	86.12 ± 3.46	91.25 ± 3.11	92.91 ± 2.78
11	18	88.14 ± 3.51	95.17 ± 1.88	95.90 ± 2.13

Mean ± SD, *n* = 3.

**Table 6:** Effect of solvent ratio on drug release in 0.1 N HCl.

Sl. number	Time (hrs)	Cumulative % drug release		
		G3	G8	G9
1	0	0.00 ± 0.00	0.00 ± 0.00	0.00 ± 0.00
2	1	21.71 ± 2.67	17.66 ± 2.67	26.39 ± 2.51
3	2	40.54 ± 4.63	34.60 ± 3.65	50.86 ± 2.40
4	3	51.68 ± 3.02	47.88 ± 2.87	56.75 ± 0.23
5	4	62.25 ± 1.58	55.32 ± 4.54	62.68 ± 1.97
6	6	69.14 ± 3.86	67.16 ± 1.87	73.93 ± 2.45
7	8	76.38 ± 1.08	71.89 ± 2.78	81.20 ± 3.34
8	10	82.10 ± 2.05	78.83 ± 1.89	84.45 ± 2.46
9	12	83.79 ± 2.87	80.51 ± 2.88	88.33 ± 0.23
10	14	86.12 ± 1.88	83.44 ± 1.45	90.37 ± 2.66
11	18	88.14 ± 3.45	84.51 ± 2.65	93.35 ± 2.71

Mean ± SD, *n* = 3.

**Table 7:** Drug release kinetics for different formulations.

Kinetic Model	Parameters	Formulation code								
		G-1	G-2	G-3	G-4	G-5	G-6	G-7	G-8	G-9
*Zero-order *	*R*	0.344	0.403	0.497	0.485	0.647	0.533	0.467	0.617	0.355
	RSS	9221	7800	6307	5485	3925	6721	7536	5095	7632
	*K*_0_	7.394	6.989	6.627	6.11	5.775	7.045	7.197	6.335	6.996

*First-order *	*R*	0.942	0.910	0.916	0.865	0.925	0.965	0.963	0.915	0.944
	RSS	0.893	1351	1271	1657	1175	653	685	1055	1287
	*K*_1_	−0.204	−0.169	−0.145	−0.115	0.107	−0.176	−0.197	−0.123	−0.162

*Higuchi *	*R*	0.915	0.927	0.943	0.947	0.965	0.956	0.945	0.962	0.934
	RSS	1637	1304	849	797	434	8.203	997	629	1092
	*k*_H_	27.344	25.747	24.336	22.457	21.001	25.813	26.433	23.102	25.794

*Hixon-Crowell *	*R*	0.846	0.816	0.823	0.785	0.853	0.897	0.886	0.844	0.846
	RSS	2672	2813	2404	2570	1842	1922	2125	1952	2726
	*k*_HC_	−0.042	−0.034	−0.037	−0.033	−0.025	−0.043	−0.046	−0.035	−0.035

*Peppas and Korsmeyer *	*R*	0.922	0.926	0.957	0.935	0.945	0.963	0.955	0.956	0.954
	RSS	11.836	959	584	642	472	504	552	683	417
	*k*	32.131	2991	28.183	25.123	21.366	30.165	32.065	23.482	33.812
	*n*	0.443	0.445	0.445	0.465	0.503	0.445	0.423	0.505	0.393

Best fit model		FO	M	PK	M	M	FO	FO	M	PK

RSS = Residual sum of squares

*k* = Release constant

*n* = Diffusion coefficient

ZO = Zero order

FO = First order

H = Higuchi

PK = Peppas Korsmeyer.

## References

[1] Hofmann A. F., Pressman J. H., Code C. F., Witztum K. F. (1983). Controlled entry of oraly administered drugs: physiological considerations. *Drug Development and Industrial Pharmacy*.

[2] Agyilirah G. A., Green M., duCret R., Banker G. S. (1991). Evaluation of the gastric retention properties of a cross-linked polymer coated tablet versus those of a non-disintegrating tablet. *International Journal of Pharmaceutics*.

[3] Baumgartner S., Kristl J., Vrečer F., Vodopivec P., Zorko B. (2000). Optimisation of floating matrix tablets and evaluation of their gastric residence time. *International Journal of Pharmaceutics*.

[4] Bulgarelli E., Forni F., Bernabei M. T. (2000). Effect of matrix composition and process conditions on casein-gelatin beads floating properties. *International Journal of Pharmaceutics*.

[5] Deshpande A. A., Shah N. H., Rhodes C. T., Malick W. (1997). Development of a novel controlled-release system for gastric retention. *Pharmaceutical Research*.

[6] Singh B. N., Kim K. H. (2000). Floating drug delivery systems: an approach to oral controlled drug delivery via gastric retention. *Journal of Controlled Release*.

[7] Timmermans J., Moes A. J. (1994). Factors controlling the buoyancy and gastric retention capabilities of floating matrix capsules: new data for reconsidering the controversy. *Journal of Pharmaceutical Sciences*.

[8] Chen J., Park K. (2000). Synthesis of fast-swelling, superporous sucrose hydrogels. *Carbohydrate Polymers*.

[9] Akiyama Y., Nagahara N., Nara E., Kitano M., Iwasa S., Yamamoto I., Azuma J., Ogawa Y. (1998). Evaluation of oral mucoadhesive microspheres in man on the basis of the pharmacokinetics of furosemide and riboflavin, compounds with limited gastrointestinal absorption sites. *Journal of Pharmacy and Pharmacology*.

[10] Chickering D. E., Jacob J. S., Desai T. A., Harrison M., Harris W. P., Morrell C. N., Chaturvedi P., Mathiowitz E. (1997). Bioadhesive microspheres: III. An in vivo transit and bioavailability study of drug-loaded alginate and poly(fumaric-co-sebacic anhydride) microspheres. *Journal of Controlled Release*.

[11] Kedzierewicz F., Thouvenot P., Lemut J., Etienne A., Hoffman M., Maincent P. (1999). Evaluation of peroral silicone dosage forms in humans by gamma-scintigraphy. *Journal of Controlled Release*.

[12] Rouge N., Allémann E., Gex-Fabry M., Balant L., Cole E. T., Buri P., Doelker E. (1998). Comparative pharmacokinetic study of a floating multiple-unit capsule, a high-density multiple-unit capsule and an immediate-release tablet containing 25 mg atenolol. *Pharmaceutica Acta Helvetiae*.

[13] Johnson F. A., Craig D. Q. M., Mercer A. D., Chauhan S. (1997). The effects of alginate molecular structure and formulation variables on the physical characteristics of alginate raft systems. *International Journal of Pharmaceutics*.

[14] Desai S., Bolton S. (1993). A floating controlled-release drug delivery system: *in vitro*-*in vivo* evaluation. *Pharmaceutical Research*.

[15] Murata Y., Sasaki N., Miyamoto E., Kawashima S. (2000). Use of floating alginate gel beads for stomach-specific drug delivery. *European Journal of Pharmaceutics and Biopharmaceutics*.

[16] Iannuccelli V., Coppi G., Bernabei M. T., Cameroni R. (1998). Air compartment multiple-unit system for prolonged gastric residence, Part I: formulation study. *International Journal of Pharmaceutics*.

[17] Ichikawa M., Watanabe S., Miyake Y. (1991). A new multiple-unit oral floating dosage system. I: preparation and in vitro evaluation of floating and sustained-release characteristics. *Journal of Pharmaceutical Sciences*.

[18] El-Kamel A. H., Sokar M. S., Al Gamal S. S., Naggar V. F. (2001). Preparation and evaluation of ketoprofen floating oral delivery system. *International Journal of Pharmaceutics*.

[19] Kawashima Y., Niwa T., Takeuchi H., Hino T., Itoh Y. (1992). Hollow microspheres for use as a floating controlled drug delivery system in the stomach. *Journal of Pharmaceutical Sciences*.

[20] Thanoo B. C., Sunny M. C., Jayakrishnan A. (1993). Oral sustained-release drug delivery systems using polycarbonate microspheres capable of floating on the gastric fluid. *Journal of Pharmacy and Pharmacology*.

[21] Wang D.-P., Yang M.-C., Wong C.-Y. (1997). Formulation development of oral controlled-release pellets of diclofenac sodium. *Drug Development and Industrial Pharmacy*.

[22] Bechgaard H. (1982). Critical factors influencing gastrointestinal absorption: what is the role of pellets?. *Acta Pharmaceutica Technologica*.

[23] Feely L. C., Davis S. S. (1989). Correlation of phenylpropanolamine bioavailability with gastrointestinal transit by scintigraphic monitoring of 111In-labeled hydroxypropylmethylcellulose matrices. *Pharmaceutical Research*.

[24] Bechgaard H., Ladefoged K. (1978). Distribution of pellets in the gastrointestinal tract. The influence on transit time exerted by the density or diameter of pellets. *Journal of Pharmacy and Pharmacology*.

[25] Rahman Z., Kohli K., Khar R. K., Ali M., Charoo N. A., Shamsher A. A. A. (2006). Characterization of 5-fluorouracil microspheres for colonic delivery. *AAPS PharmSciTech*.

[26] Zinutti C., Barberi-Heyob M., Hoffman M., Maincent P. (1998). In-vivo evaluation of sustained release microspheres of 5-FU in rabbits. *International Journal of Pharmaceutics*.

[27] Haznedar S., Dortunç B. (2004). Preparation and in vitro evaluation of Eudragit microspheres containing acetazolamide. *International Journal of Pharmaceutics*.

[28] Paulo C., Jose M. S. L. (2001). Modeling and comparison of dissolution profiles. *European Journal of Pharmaceutical Sciences*.

[29] Lee J.-H., Park T. G., Choi H.-K. (1999). Development of oral drug delivery system using floating microspheres. *Journal of Microencapsulation*.

[30] Subrahmanyam C. V. S. (2002). *Textbook of Physical Pharmaceutics*.

[31] Soppimath K. S., Kulkarni A. R., Aminabhavi T. M. (2001). Development of hollow microspheres as floating controlled-release systems for cardiovascular drugs: preparation and release characteristics. *Drug Development and Industrial Pharmacy*.

[32] Patel A., Ray S., Thakur R. S. (2006). In-vitro evaluation and optimization of controlled release floating drug delivery system of metformin hydrochloride. *DARU*.

[33] Fell J. T., Digenis G. A. (1984). Imaging and behaviour of solid oral dosage forms in vivo. *International Journal of Pharmaceutics*.

[34] Bomma R., Naidu R. S., Yamsani M., Veerabrahma K. (2009). Development and evaluation of gastroretentive norfloxacin floating tablets. *Acta Pharmaceutica*.

